# Radiotherapy-Induced Cardiotoxicity: The Role of Multimodality Cardiovascular Imaging

**DOI:** 10.3389/fcvm.2022.887705

**Published:** 2022-07-28

**Authors:** Tomaž Podlesnikar, Boštjan Berlot, Jure Dolenc, Katja Goričar, Tanja Marinko

**Affiliations:** ^1^Department of Cardiology, University Medical Centre Ljubljana, Ljubljana, Slovenia; ^2^Department of Cardiac Surgery, University Medical Centre Maribor, Maribor, Slovenia; ^3^Pharmacogenetics Laboratory, Institute of Biochemistry and Molecular Genetics, Faculty of Medicine, University of Ljubljana, Ljubljana, Slovenia; ^4^Department of Radiotherapy, Institute of Oncology Ljubljana, Ljubljana, Slovenia; ^5^Faculty of Medicine, University of Ljubljana, Ljubljana, Slovenia

**Keywords:** radiotherapy, cardiotoxicity, heart failure, multimodality cardiovascular imaging, echocardiography, cardiovascular magnetic resonance

## Abstract

Radiotherapy (RT) is one of the pillars of cancer therapy. High-dose radiation exposure on the thorax is mainly used in the context of adjuvant RT after breast surgery, in lung and esophageal cancer, and as a complement to systemic treatment in lymphoma. Due to the anatomical proximity, the heart inevitably receives some radiation that can result in acute and chronic cardiotoxicity, leading to heart failure, coronary artery disease, pericardial and valvular heart disease. Current evidence suggests there is no safe radiation dose to the heart, which poses a need for early recognition of RT-induced cardiac injury to initiate cardioprotective treatment and prevent further damage. Multimodality cardiac imaging provides a powerful tool to screen for structural and functional abnormalities secondary to RT. Left ventricular ejection fraction, preferably with three-dimensional echocardiography or cardiovascular magnetic resonance (CMR), and global longitudinal strain with speckle-tracking echocardiography are currently the key parameters to detect cardiotoxicity. However, several novel imaging parameters are tested in the ongoing clinical trials. CMR parametric imaging holds much promise as T1, T2 mapping and extracellular volume quantification allow us to monitor edema, inflammation and fibrosis, which are fundamental processes in RT-induced cardiotoxicity. Moreover, the association between serum biomarkers, genetic polymorphisms and the risk of developing cardiovascular disease after chest RT has been demonstrated, providing a platform for an integrative screening approach for cardiotoxicity. The present review summarizes contemporary evidence of RT-induced cardiac injury obtained from multimodality imaging—echocardiography, cardiovascular computed tomography, CMR and nuclear cardiology. Moreover, it identifies gaps in our current knowledge and highlights future perspectives to screen for RT-induced cardiotoxicity.

## Introduction

Radiotherapy (RT) is one of the pillars of cancer therapy (1). It has been used alone or in combination with surgery and systemic oncological treatments for a wide range of malignancies to maximize tumor control and quality of life while minimizing toxicity and preserving the organs. The side effects of RT depend on the anatomic area of treatment and are related to treatment factors such as the cumulative dose, dose per fraction, proximity of sensitive tissues and organs, and the effect of other cancer treatments, such as surgery and chemotherapy. High-dose radiation exposure on the thorax and the heart is mainly used in the context of adjuvant RT after breast surgery, RT of lung and esophageal cancer, and as a complement to systemic treatment in lymphoma (2). Older RT techniques, used to treat patients with thoracic malignancies caused an increase in cardiovascular morbidity and mortality in long-term follow-up (3–5).

**FIGURE 1:**
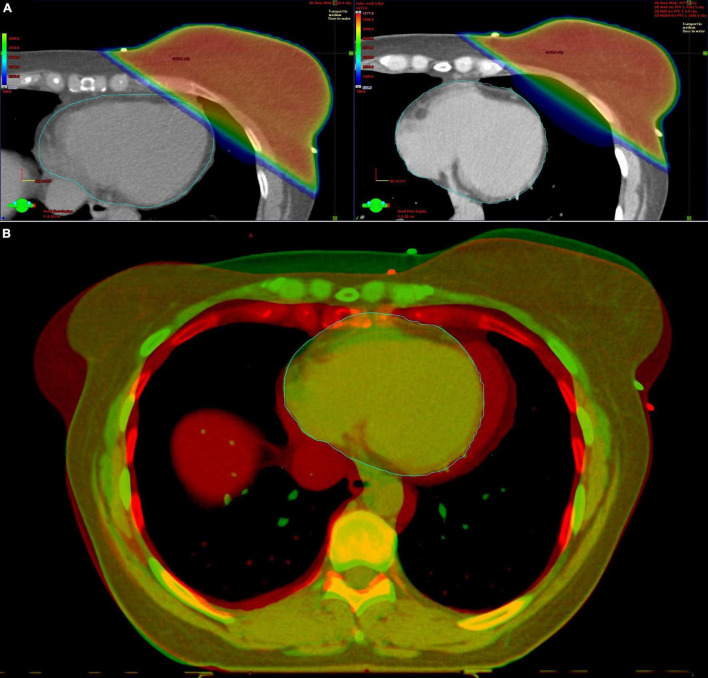
Deep inspiration breath hold (DIBH) radiotherapy for left-sided breast cancer. **(A)** Axial computed tomography slices with radiotherapy plan at the same level of the breast in free breathing (left) and DIBH (right). The distance between the heart and the breast is greater in DIBH, minimizing the radiation of the heart. **(B)** Fusion image of the free breathing (red color) and DIBH (green color) computed tomography scan.

Awareness that delayed effects on the heart may lower the therapeutic benefits from RT has an important impact on modern cardio-oncology clinical practice. To minimize the radiation to the surrounding tissues, treatment plans for patients with Hodgkin lymphoma have evolved from extended-field to involved-field and then to involved-site/involved-node RT (6). Furthermore, there have been significant technologic advancements in the radiation delivery techniques, with original 2-dimensional (2D) treatment planning being replaced by much more conformal approaches, such as 3-dimensional conformal RT (3DCRT), intensity-modulated RT (IMRT), and proton beam therapy (6). Cardiac sparing techniques, such as deep inspiration breath hold RT, have been introduced to minimize radiation dose to the heart, especially in patients with left-sided breast cancer (Figure 1) (7). However, due to the demand for covering the tumor bed lying close to the heart with a high radiation dose, some radiation dose to the heart will always remain an issue.

The present review focuses on the current evidence of RT-induced cardiac injury obtained from multimodality imaging—echocardiography, cardiovascular computed tomography (CT), cardiovascular magnetic resonance (CMR), and nuclear cardiology. In addition, it identifies gaps in our knowledge and highlights future perspectives to diagnose and monitor RT-induced cardiotoxicity.

## Pathophysiology of Radiotherapy-Induced Cardiotoxicity

**FIGURE 2:**
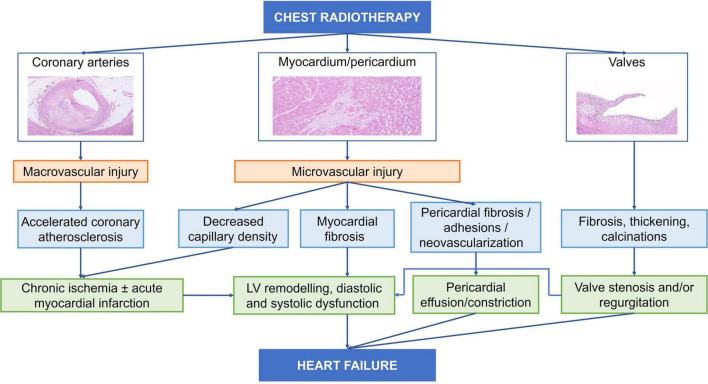
Pathophysiology of radiotherapy-induced cardiotoxicity.

Radiation damage is characterized by acute and chronic changes in the myocardium, pericardium, coronary vasculature, valvular apparatus and the conduction system. The pathophysiology shares several common pathways and mechanisms that ultimately lead to a progressive decline in left ventricular (LV) diastolic and systolic function and cause symptoms of heart failure (Figure 2).

Central to the RT-induced myocardial injury is the vascular endothelial cell damage, occurring within minutes of ionizing radiation and leading to an acute inflammatory response (8). The acute pro-inflammatory milieu is a potent initiator of fibrosis (9). Fibroblasts are recruited from many different sources, resulting in collagen deposition and endothelial cell proliferation. In addition, radiation changes the biology of pro-fibrotic cells, inducing premature differentiation of fibroblasts, which are five to eight times more active in the production of interstitial collagens than progenitor fibroblasts (10). The inflammatory pathway is likely the predominant pro-fibrotic mediator, but other mechanisms like chronic oxidative stress and altered gene expression contribute significantly (8, 11). Progressive fibrosis of the myocardium leads to a decrease in capillary density and increased myocardial stiffness, resulting first in diastolic and later in systolic myocardial dysfunction.

Acute inflammatory changes involving the pericardium may cause acute pericardial effusion (12). Ensuing local ischemia leads to tortuous and permeable neovascularization, leading to accumulation of fibrin-rich pericardial exudate (8). In addition, fibrosis of the venous and lymphatic channels in the heart decreases the ability to drain extracellular fluid. Fibrinous exudates are later replaced by fibroblasts laying down collagen, leading to long-term fibrosis of the pericardium. These changes can manifest as chronic pericardial effusion and constrictive pericarditis (2).

Radiation exposure affects as well the coronary vascular bed. The initiation of RT-induced injury is similar to that of the myocardium—the radiation damages the endothelial cells, which respond with inflammatory markers and adhesion molecules to recruit peripheral leukocytes (8). Once monocytes enter the subendothelial space, they transform into activated macrophages, ingest lipids and form fatty streaks in the intima. Late proliferation of myofibroblasts can further facilitate the growth of these luminal-narrowing lesions. In general, the pathologic changes observed after RT are morphologically similar to the atherosclerotic disease. However, lesions tend to be longer, more concentric and typically affect the ostia of major coronary arteries (13).

The hallmarks of RT-induced valvular heart disease are thickening, fibrosis and calcification of the valve apparatus (2). RT predominantly affects the left-sided valves and regurgitant lesions are more common than stenotic. However, the mechanism of valvular injury is less well characterized. No changes indicative of chronic inflammation or neovascularization were found on the valve leaflets with histopathology, suggesting that another RT-related mechanism drives the valvular pathology (14).

## Multimodality Cardiovascular Imaging in Radiotherapy-Induced Cardiac Injury

### Echocardiography

Echocardiography is the first-line imaging modality to assess cardiotoxicity in cancer patients. Owing to its wide availability and accessibility, low-risk profile, and ability to assess systolic and diastolic function, valvular pathophysiology, and pericardial disease, it is ideal technique for screening and diagnosing RT-related cardiovascular toxicity (6).

Traditionally, LV ejection fraction (LVEF) is the gold standard to assess global systolic function. More than 10 percentage points decrease in LVEF to a value below the lower limit of normal [54% for women and 52% for men according to the current European and American recommendations (15)] has been recommended as a cutoff to diagnose cardiotoxicity (16, 17). The 2D biplane Simpson method is the most commonly employed technique, however, it may struggle with image quality (common after RT or mastectomy) and has relatively high inter- and intra-observer variability (18, 19). LV contrast agents may improve image quality and should be used whenever two or more LV segments are not adequately visualized in the apical views (15, 20). Three-dimensional (3D) echocardiography has better reproducibility (19) and should be used in LVEF calculation when available (16, 17).

**FIGURE 3:**
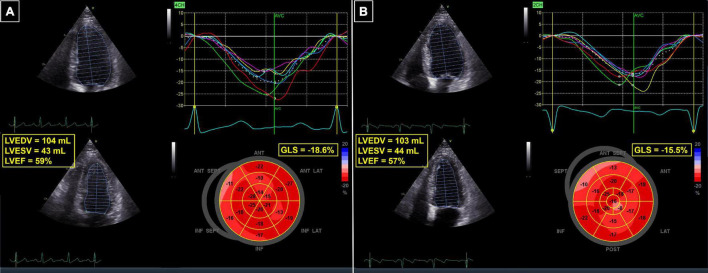
62-year-old patient with breast cancer undergoing chemotherapy with anthracyclines and radiotherapy. **(A)** Baseline echocardiogram, showing normal LVEF and GLS. **(B)** Follow-up echocardiogram 3 months after completion of therapy—the LVEF was still normal, but a 17% relative reduction in GLS revealed cardiotoxicity. GLS, global longitudinal strain; LVEDV, left ventricular end-diastolic volume; LVEF, left ventricular ejection fraction; LVESV, left ventricular end-systolic volume.

Myocardial deformation imaging with tissue Doppler and 2D speckle-tracking echocardiography provides incremental value for the assessment of LV function in cancer patients. Global longitudinal strain (GLS) is the most commonly used deformation parameter and a 15% relative reduction in GLS or absolute reduction below −18% suggest cardiotoxicity (16, 17). Several studies have shown that a reduction in GLS is more sensitive than LVEF to evaluate subtle myocardial dysfunction associated with modern RT strategies (Figure 3) (21–24). Erven et al. (21) studied tissue Doppler strain imaging in 75 breast cancer patients (51 left-sided and 24 right-sided) before RT, immediately after RT and at 8 and 14 months after RT (all patients also received anthracycline- and taxane-based chemotherapy before RT). In contrast to the LVEF, which did not change over time, GLS in patients with left-sided breast cancer declined immediately after RT and remained impaired during follow-up (−17.5 ± 1.9% after RT, −16.6 ± 1.4% at 8 months, and −17.7 ± 1.9% at 14 months compared to −19.4 ± 2.4% before RT, *P* < 0.01). No decline in GLS was observed in patients with right-sided breast cancer. When comparing the segmental strain values, the strain of the anterior LV wall receiving the highest radiation dose was significantly reduced, while the strain of the inferior LV wall receiving the lowest radiation dose did not change. Similar findings were confirmed in breast cancer patients receiving RT without concomitant chemotherapy (22–25). Tuohinen et al. (22) observed a decrease in GLS with speckle-tracking echocardiography as early as 3 days after completion of RT. The changes persisted at 3-year follow-up, when a relative reduction in GLS of more than 15% was present in 27% of patients. Interestingly, while studies with shorter follow-up (up to 1 year after RT) found no changes in LVEF (23, 24), the authors observed as well a significant decline in LVEF (59% at 3-year follow-up vs. 65% at baseline, *P* < 0.001) (22).

Furthermore, the segmental strain analysis demonstrated that variability in dose distribution across the heart plays an important role in cardiotoxicity. Breast cancer patients had the greatest reductions in myocardial strain in the anterior wall followed by the anteroseptal and the anterolateral walls with a base-to-apex gradient and this was correlated with the segmental distribution of the received radiation dose (25). Moreover, multi-layer strain analysis showed that RT primarily affects the endocardial layer (26). However, it should be noted that not all studies have confirmed these findings. Yu et al. (27) found no change in GLS in breast cancer patients 6 months after RT and Heggemann et al. (28) found only a transient decrease in GLS at 6 and 12 months, followed by an increase to baseline values at 24 months after RT.

A population-based study has shown that the predominant form of heart failure after contemporary RT for breast cancer is heart failure with preserved ejection fraction (LVEF ≥ 50%), implying impaired LV diastolic function (29). This has been linked with autopsy findings of increased myocardial fibrosis (14). However, most echocardiographic studies evaluating RT toxicity in breast cancer patients failed to show significant impairment in traditional echocardiographic diastolic function parameters, i.e., mitral inflow and tissue Doppler annular velocities (21–23). Despite the lack of clear prognostic value, the joint European and American recommendations advocate comprehensive assessment of diastolic function in patients receiving cancer therapy (20, 30). Moreover, contemporary studies have shown impaired early and late global diastolic strain rate 6 weeks after RT (31) and impaired early diastolic strain rate in apical and anteroseptal segments at 3-year follow-up (32). Changes in the apical early diastolic strain rate were present even in patients with preserved GLS and were independently associated with RT dose and cardiovascular comorbidities (32).

Right ventricular (RV) abnormalities may also occur in oncological patients, however, much less is known about RV than LV impairment (20). Christiansen et al. (33) studied 246 childhood cancer survivors (malignant lymphoma or acute lymphoblastic leukemia) 21.7 ± 8.1 years after diagnosis, who had been exposed to chemotherapy, mediastinal RT, or both. Compared with 211 matched controls, there were no differences in RV diastolic dimensions but the mean measures of RV function were all lower in the survivors group: fractional area change (44.5 vs. 48.6%, *P* < 0.001), tricuspid annular plane systolic excursion (2.24 vs. 2.49 cm, *P* < 0.001), peak systolic tricuspid annular velocity (12.1 vs. 13.0 cm/s, *P* = 0.001), and RV free wall strain (−26.5 vs. −28.4%, *P* < 0.001). Signs of RV systolic dysfunction were 3 times more often in patients with concomitant LV dysfunction. These findings were confirmed in another cohort of 274 adult lymphoma survivors examined with echocardiography 13 ± 6 years after diagnosis (34). After multivariate adjustments, all parameters of RV systolic function were impaired in patients treated with high-dose cardiac RT compared with the patients receiving only chemotherapy. However, RV dysfunction was less common than LV dysfunction (6.2% vs. 30.8%, respectively; *P* < 0.001) and the majority of patients with RV dysfunction were asymptomatic, leading the authors to conclude that RV dysfunction is most likely of subclinical importance. Impaired global and free wall RV strain was observed in 128 patients with non-small lung cell carcinoma 6 months after receiving chemo- and RT (35). Both strain indices correlated with the mean radiation dose and in multivariate analysis RV free wall strain was an independent predictor of all-cause mortality. Impaired RV function after RT was also demonstrated in patients with breast cancer (36).

Echocardiography is also an excellent method to diagnose valvular heart disease. The echocardiographic characteristics of RT-induced valve disease include fibrosis and calcification of the aortic root, aortic valve annulus and leaflets, mitral valve annulus, and the base and mid-portions of the mitral valve leaflets (2, 20). Typically, the mitral valve tips and the commissures are less affected. Several studies have found high prevalence of asymptomatic valvular heart disease following high-dose mediastinal irradiation for Hodgkin lymphoma (4, 37, 38). Mild or greater aortic regurgitation was the most prevalent condition, occurring in 38–60% of patients (37, 38). Pericardial disease after modern RT is nowadays rare, however, echocardiography is well equipped to diagnose pericardial thickening, pericardial effusion and characteristic hemodynamic features of constrictive pericarditis (2, 20). Furthermore, stress echocardiography (ether exercise, dobutamine or vasodilator) is an important method to diagnose functionally significant coronary artery disease as a consequence of RT (2, 20).

Table 1 summarizes the most important echocardiographic studies to detect RT-induced cardiotoxicity.

**TABLE 1:** Echocardiographic studies to detect RT-induced cardiotoxicity.

Study	Cancer type	Therapy	No. of patients	Main findings
Erven et al. (21)	Breast cancer	RT + CTx	75	GLS in left-sided breast cancer declined immediately after RT and remained impaired during 14-month follow-up. No change in LVEF.
Tuohinen et al. (22)	Breast cancer	RT	81	A significant reduction in LVEF and GLS 3 years after RT. 27% of patients had > 15% relative reduction in GLS.
Trivedi et al. (23)	Breast cancer	RT	40	A significant reduction in GLS and LV S’ velocity at 12 months after RT, no change in LVEF.
Walker et al. (24)	Breast cancer	RT	79	A > 10% relative reduction in GLS 6 months after RT was associated with RT dose (LV volume exposed to ≥ 20 Gy).
Trivedi et al. (25)	Breast cancer	RT	61	Impaired segmental longitudinal strain correlated with segmental distribution of the received radiation dose.
Walker et al. (26)	Breast cancer	RT	64	Longitudinal strain after RT decreased primarily in the endocardial layer.
Yu et al. (27)	Breast cancer	RT + CTx	47	No change in GLS, GCS, and GRS at 6 months after RT.
Heggemann et al. (28)	Breast cancer	RT ± CTx	49	A decrease in GLS at 6 and 12 months after RT, followed by a return to baseline values at 24 months after RT.
Saiki et al. (29)	Breast cancer	RT ± CTx	170	The predominant form of HF after contemporary RT was HFpEF. The relative risk of HFpEF increased with increasing cardiac radiation exposure.
Sritharan et al. (31)	Breast cancer	RT	40	Impaired early and late global diastolic strain rate 6 weeks after RT. No change in traditional diastolic parameters.
Tuohinen et al. (32)	Breast cancer	RT	60	Impaired early global diastolic strain rate in apical and anteroseptal segments 3 years after RT, even in patients with preserved GLS.
Christiansen et al. (33)	Childhood cancer	RT and/or CTx	246	Impaired RV systolic function (FAC, TAPSE, S’ velocity, free wall strain) at 21.7 years after therapy compared to matched controls.
Murbraech et al. (34)	Lymphoma	CTx ± RT	274	Impaired RV systolic function (FAC, TAPSE, S’ velocity, global and free wall strain) at 13 ± 6 years after therapy among patients treated with high-dose cardiac RT compared to patient receiving chemotherapy alone.
Chen et al. (35)	Non−small cell lung cancer	RT + CTx	128	A significant reduction in RV global and free wall strain 6 month after therapy. RV free wall strain was independent predictor of all-cause mortality.
Tuohinen et al. (36)	Breast cancer	RT	49	A significant reduction in TAPSE immediately after RT.

*CTx, chemotherapy; FAC, fractional area change; GCS, global circumferential strain; GLS, global longitudinal strain; GRS, global radial strain; HF, heart failure; HFpEF, heart failure with preserved ejection fraction; LV, left ventricular; LVEF, left ventricular ejection fraction; RT, radiotherapy; RV, right ventricular; TAPSE, tricuspid annular plane systolic excursion.*

### Cardiovascular Computed Tomography

Cardiovascular CT provides detailed cross-sectional anatomical imaging of the chest with a powerful depiction of coronary, pericardial, myocardial, and vascular anatomy. It includes non-contrast CT, highly sensitive for calcified tissues, and CT angiography (CTA), where cardiac cavities and vessels are distinguished from the surrounding tissues by contrast medium opacification. The acquisition and reconstruction of images must be synchronized to the ECG to obtain robust motion-free images of moving structures like coronary arteries. The main disadvantages of CT are the ionizing radiation (effective dose in the range of < 1–12 mSv, depending on the scanning protocol), the need for iodine-containing contrast media, and the susceptibility to arrhythmias and breath-hold motion artifacts (39–41).

Cardiovascular CT can be used to assess cardiac chamber dimensions, mass, LV, and RV ejection fraction with good accuracy and reproducibility compared to CMR (42). The temporal resolution in the range of 100 ms allows the evaluation of regional wall motion abnormalities (43), but at the expense of higher radiation dose needed to acquire the whole cardiac cycle. Thus, cardiovascular CT is not the first-choice imaging technique for serial assessment of LV and RV function in suspected RT-induced heart failure. It is used only when echocardiography or CMR are not available.

**FIGURE 4:**
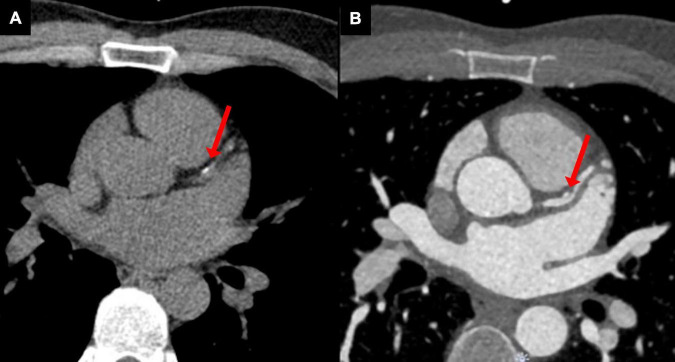
Young patient treated with radiotherapy for Hodgkin lymphoma in childhood with premature atherosclerosis. **(A)** A non-contrast computed tomography scan, used for the assessment of coronary artery calcium score, showed calcinations in the proximal left anterior descending coronary artery (red arrow). **(B)** Coronary computed tomography angiography revealed a non-obstructive vulnerable plaque (red arrow).

Cardiovascular CT is a powerful tool to screen for coronary artery disease. To identify calcium deposits in coronary vasculature and to assess the coronary artery calcium (CAC) score, a low radiation dose ECG-gated non-contrast CT scan is performed (Figure 4A) (44). A CAC score predicts atherosclerotic cardiovascular events in a graded fashion, independent of other risk factors, such as age, sex, and ethnicity, and it can identify patients who may benefit from primary prevention measures (45, 46). Non-ECG gated chest CT is routinely performed for chest radiation planning and cancer staging. Although its diagnostic accuracy is lower than the triggered examination, it can reliably detect CAC burden (47, 48). In a population of 939 female breast cancer patients treated with RT, a CAC score ≥ 100 on radiation planning CT was associated with increased incidence of acute coronary events over a 9-year follow-up compared to patients with a CAC score of 0 (hazard ratio 4.95, 95% confidence interval 1.69–14.53, *P* = 0.004) (47). The relationship was significant even after correcting for confounding factors such as age, cardiovascular risk factors, and mean heart dose. CAC score was a better predictor of all-cause mortality and cardiac events than Framingham risk score in another study with breast cancer patients (49). The international cardio-oncology society consensus statement on therapeutic radiation recommends reviewing available chest CT scans (acquired before or after RT) for the presence of coronary and aortic calcifications to improve cardiovascular risk stratification and guide therapy (statin and/or aspirin use) (50). However, the CAC score threshold (>0, > 10, > 100) that should prompt initiation of cardioprotective therapy is not known.

While CAC scans are limited to identifying coronary calcium deposits, coronary CTA depicts complete coronary anatomy, including non-calcified plaques, but at the expense of slightly higher radiation dose and the use of contrast agents (Figure 4B). Coronary CTA has an excellent negative predictive value in detecting coronary artery disease (51). CTA guided patient care was superior to standard care in reducing cardiovascular death and myocardial infarction among patients with stable chest pain during 5-year follow-up (52). It is unknown whether patients after RT have a higher risk of non-calcified plaque, in which case coronary CTA would be preferred over CAC for screening (50). The international cardio-oncology society recommends screening for coronary artery disease either with CAC, coronary CTA or functional stress testing in patients with prior chest irradiation without documented atherosclerosis at 5-year intervals (50). In an asymptomatic patient already on optimal preventive therapy, repeated screening is not warranted as it would unlikely change the management (50).

Cardiovascular CT is a valuable tool to identify pericardial complications of chest RT (53). Normal pericardium appears on CT as a thin hyperdense layer less than 3 mm thick. Since it is surrounded by low attenuated fat, pericardium can be visualized even without contrast. However, contrast media are essential in differentiating pericardial thickening from small effusion and to identify inflammation. Although rare nowadays, thickening of the pericardium with increased attenuation and pericardial effusion are the hallmarks of acute radiation-induced pericarditis. CT can distinguish between serous transudates [typically with attenuation < 10 Hounsfield units (HU)], non-serous exudates with attenuation 20–40 HU and hemorrhagic effusions with attenuation 40–60 HU (54). With specific CT findings such as pericardial thickening, calcifications, narrowing or tubular deformation of the RV, large atria, and venous congestion, CT can raise suspicion of constrictive pericarditis.

Cardiovascular CT has a vital role in evaluating aortic, valvular and myocardial calcifications. In patients requiring cardiac surgery, preoperative assessment of mediastinal fibrosis and aortic calcifications is essential to determine the suitability of a surgical approach and the site of aortic cross-clamping (55). Significant valvular and/or annular calcifications may present high-risk features for surgical or transcatheter valve procedures. The European guidelines for the management of valvular heart disease favor transcatheter aortic valve implantation to surgical valve replacement in patients with sequelae of chest radiation (56). In patients undergoing coronary artery bypass graft surgery after RT involving the subclavian artery and/or internal mammary artery (i.e., patients with breast cancer receiving regional nodal irradiation) and in whom the latter is being contemplated as a bypass graft, CTA is recommended to evaluate subclavian artery stenosis and internal mammary artery patency (50).

### Cardiovascular Magnetic Resonance

CMR is a relatively new cardiac imaging technique that has witnessed major technical advancements, application to a broad range of cardiovascular diseases, as well as incorporation into consensus statements and clinical practice guidelines in the last 2 decades (57). CMR combines the advantages of tomographic and functional imaging modalities. Due to its high spatial and temporal resolution, excellent contrast between myocardium and blood pool and high reproducibility it has evolved as a gold standard for the assessment of ventricular volumes and systolic function. Furthermore, CMR allows myocardial tissue characterization, blood flow analysis, myocardial perfusion assessment, and provides information on extracardiac findings. This makes CMR a very useful technique to assess cardiotoxicity after cancer therapy. The main limitations to wider clinical application of CMR are its limited availability and relatively high cost. Furthermore, it is contraindicated in patients with severe renal impairment, metal implants and medical devices (e.g., certain implantable cardioverter-defibrillators, pacemakers), claustrophobia, and anxiety attacks. Moreover, the inability to carry out repeated breath holds and the presence of arrhythmias might represent additional problems to acquire high-quality data.

#### Cardiovascular Magnetic Resonance Techniques to Evaluate Cardiotoxicity

T1- and T2-weighted anatomical images in the axial, coronal, and sagittal planes provide basic morphological data regarding cardiothoracic structures. In RT-induced cardiac disease pericardial thickening or pericardial effusion might be found. In addition, extracardiac findings of interest might be diagnosed (e.g., metastatic foci).

**FIGURE 5:**
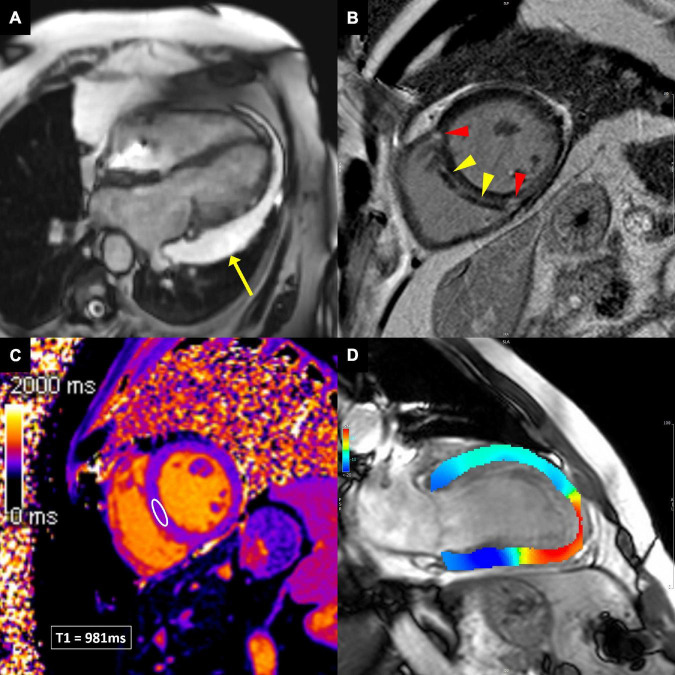
Cardiovascular magnetic resonance techniques to evaluate cardiotoxicity. **(A)** Balanced steady-state free precession cine image in breast cancer patient demonstrating large pericardial effusion next to the lateral wall of the left ventricle (yellow arrow). **(B)** Late gadolinium enhancement image, showing linear midwall myocardial fibrosis (yellow arrowheads), indicating non-ischemic dilated cardiomyopathy. In addition, the anterior and inferior right ventricular insertion point fibrosis is present (red arrowheads), which is a non-specific finding. **(C)** Native T1 image, allowing T1 measurements in any desired myocardial area of interest (white ellipse in the ventricular septum). **(D)** Feature-tracking strain image, showing impaired longitudinal strain in the apex and apical inferior wall (red color), consistent with apical myocardial infarction.

Cine balanced steady-state free precession images in multiple views allow essential functional and anatomical evaluation of cardiac chambers, including measurements of LV and RV volumes, ejection fraction and mass, as well as the assessment of segmental wall motion abnormalities, valve anatomy and pericardium (Figure 5A) (58).

T2-weighted short tau inversion recovery and bright blood T2-weighted sequences are most commonly used to evaluate myocardial edema (58).

Late gadolinium enhancement (LGE) is considered the gold standard for non-invasive evaluation of myocardial fibrosis (58). About 10–20 min after gadolinium-based contrast agent injection, the difference in contrast washout between the normal and abnormal myocardium can be visualized so that the normal myocardial appears black and areas of fibrosis appear white (58). However, LGE is sensitive to any kind of extracellular volume expansion and delayed contrast washout might as well be present in myocardial edema or other extracellular deposits (e.g., amyloidosis). The pattern and distribution of LGE can imply ischemic or non-ischemic etiology of myocardial disease (Figure 5B) (59, 60).

Novel CMR sequences for detection of myocardial inflammation, edema and fibrosis allow quantification of changes in T1 and T2 relaxation times and computation of extracellular volume (ECV) (Figure 5C) (60, 61). These techniques can detect diffuse processes involving the entire myocardium, unlike the standard edema and LGE techniques, which rely on signal intensity differences between normal and diseased myocardium. Furthermore, direct quantification improves diagnostic confidence and intra- and inter-observer variability (62). In the setting of myocardial inflammation/edema, T2 mapping is the most sensitive parameter (62). Increased native T1 values can reflect both edema and fibrosis, while ECV is the most sensitive parameter to detect myocardial fibrosis.

Several CMR techniques have been developed in the past to analyze myocardial deformation, e.g., myocardial tagging, displacement encoding with stimulated echoes, strain-encoded imaging. Their main shortcoming was the need for special scan sequences. Recently, feature-tracking CMR has been developed, which is based on tissue tracking algorithm applied to standard cine images during postprocessing, similar to speckle-tracking echocardiography (Figure 5D) (63). LV strain (and strain rate) can be measured in all three directions of cardiac motion—longitudinal, circumferential, and radial. Feature-tracking CMR has been increasingly utilized in various cardiac diseases and has shown incremental prognostic value to common clinical and CMR imaging risk factors, including LVEF and LGE (64–67).

#### Radiotherapy-Induced Cardiotoxicity With Cardiovascular Magnetic Resonance—Current Evidence

CMR has been increasingly utilized in cardio-oncology research trials (Table 2). van der Velde et al. (68) studied 80 lymphoma survivors at 20 ± 8 years after mediastinal RT (median dose 36 Gy, 88% of patients also received anthracyclines), and results were compared with 40 healthy control subjects matched for age and sex. Significantly lower LVEF (53 ± 5% vs. 60 ± 5%; *P* < 0.001) and LV mass (47 ± 10 g/m^2^ vs. 56 ± 8 g/m^2^; *P* < 0.001) were found in lymphoma survivors. Global LV longitudinal, circumferential and radial strain with feature-tracking CMR were reduced compared to healthy controls. LGE was present in 11% of the survivors (5% myocardial infarction pattern, 6% non-ischemic pattern) and the native T1, indicative of diffuse myocardial fibrosis, was significantly increased compared to healthy controls (980 ± 33 ms vs. 964 ± 25 ms; *P* = 0.007).

**TABLE 2:** CMR studies to detect RT-induced cardiotoxicity.

Study	Cancer type	Therapy	No. of patients	CMR technique	Main findings
van der Velde et al. (68)	Lymphoma	RT ± CTx	80	Cine, LGE, feature-tracking, T1 mapping	Reduced LVEF, LV mass, GLS, GCS, GRS, higher native T1 and 11% prevalence of LGE at 20 ± 8 years after therapy.
Heggemann et al. (28)	Breast cancer	RT ± CTx	49	Cine, LGE	A decrease in LVEF at 6 months after RT, followed by a return to baseline at 24 months after RT. No LGE.
Bergom et al. (69)	Breast cancer	RT + CTx	20	Cine, LGE, feature-tracking, T1 mapping	No correlations between whole heart doses and LVEF, LV dimensions, LV mass, GLS and ECV 8.3 years after therapy.
Umezawa et al. (70)	Esophageal cancer	RT ± CTx	24	LGE	LGE (mainly non-ischemic) was detected in 50% of patients at 23.5 months after RT. LGE was present in 15.4 and 21.2% myocardial segments exposed to > 40 Gy and > 60 Gy, while no LGE was found outside radiation field.
Tahir et al. (71)	Breast cancer	RT or CTx	66	Cine, LGE, feature-tracking, T1, T2 mapping	No changes in conventional parameters, strain, T1 and T2 values immediately after RT and at 1-year follow-up in the RT group. A transient increase in native T1 and T2 values at 2 ± 2 weeks in the CTx group.
Foulkes et al. (72)	Childhood cancer	CTx ± RT	20	Cine, feature tracking, exercise CMR	Reduced cardiac reserve and attenuated stroke volume increase on exercise CMR in 60% of patients 4.4 years after diagnosis.

*CMR, cardiovascular magnetic resonance; ECV, extracellular volume; LGE, late gadolinium enhancement. Other abbreviations as in Table 1.*

Heggemann et al. (28) have studied 49 patients with left-sided breast cancer treated with 3DCRT or IMRT. Twenty patients also received concomitant chemotherapy. The mean heart dose was 4.5 ± 2.4 Gy for 3DCRT and 12.9 ± 3.9 Gy for IMRT and the heart volumes receiving > 40 Gy were 2.6 and 1.3%, respectively. Patients underwent serial CMR evaluation before RT and at 6, 12, and 24 months after RT. A transient decrease in LVEF was observed on 6-month CMR (59% vs. 63% at baseline, *P* = 0.005), resolving at 24 months. No wall motion abnormalities and no LGE were found. Similarly, Bergom et al. (69) find no correlation between the mean heart irradiation dose (4.8 Gy, range 1.1–11.2 Gy) and CMR-derived LV dimensions, LVEF, LV mass, GLS and ECV in 20 breast cancer patients at 8.3 years after anthracycline chemotherapy and regional nodal irradiation with 3DCRT.

Umezawa et al. (70) have studied myocardial fibrosis with LGE CMR in 24 esophageal cancer patients treated with RT. In all patients LV was partially involved in the irradiation field and at a median of 2 years after curative RT (median total dose 66 Gy) LGE was found in 50%. LGE was mid-myocardial (indicating non-ischemic injury) in 11 patients and subendocardial (indicating ischemic injury) in 1 patient. In myocardial segments exposed to > 40 Gy and > 60 Gy irradiation dose LGE was detected in 15.4 and 21.2%, respectively, while no LGE was found in LV segments outside the radiation field. The study unequivocally demonstrated the association between myocardial tissue injury and high heart irradiation doses.

Tahir et al. (71) studied cancer therapy-related cardiotoxicity in 39 breast cancer patients who received anthracycline chemotherapy and 27 patients who underwent left-sided RT (mean heart dose was 2 ± 2 Gy). Patients receiving anthracycline chemotherapy showed a transient increase in native T1 and T2 values at 2 ± 2 weeks after completion of chemotherapy compared to baseline (1,293 ± 34 ms vs. 1,244 ± 29 ms for native T1, *P* < 0.001; and 48 ± 3 ms vs. 45 ± 3 ms for T2, *P* < 0.001), indicating myocardial edema. Both native T1 and T2 returned to baseline values approximately 1 year after completion of therapy. In contrast, patients undergoing left-sided RT did not show increased T1 or T2 values neither immediately after RT nor at 1-year follow-up. Both groups also had normal ECV values at 1-year follow-up.

Foulkes et al. (72) studied 20 pediatric cancer survivors treated with anthracycline chemotherapy with or without concomitant RT with exercise CMR using a CMR compatible ergometer 4.4 years after diagnosis. Twelve (60%) patients had reduced peak oxygen consumption (VO_2_; defined as < 85% age-predicted values) and despite having similar resting cardiac function in terms of LVEF and GLS (with feature-tracking) compared to their counterparts with normal peak VO_2_, they demonstrated reduced cardiac reserve on exercise CMR. Their maximum cardiac index was less than in patients with normal peak VO2 (6.8 ± 1.2 L/min/m^2^ vs. 9.0 ± 1.6 L/min/m^2^, respectively; *P* = 0.003) and increased mainly due to the increase in the heart rate, while their maximum stroke volume index was markedly reduced (44 ± 9 mL/m^2^ vs. 60 ± 15 mL/m^2^, respectively; *P* = 0.007).

### Nuclear Cardiology

Nuclear cardiology imaging techniques, including multigated radionuclide angiography, single-photon emission computed tomography (SPECT) and positron emission tomography (PET), have been used to assess cardiac disease in cancer survivors (16, 73). ECG-gated protocols, either by myocardial perfusion or blood pool techniques, can give accurate and reproducible measurements of cardiac chamber volumes and ejection fraction and are still considered a reference standard for the assessment of LV function in the setting of cardiotoxicity (16, 74). The recently introduced SPECT with cadmium-zinc-telluride cameras, which allow superior spatial resolution with a low-dose radiation, has demonstrated usefulness in LV diastolic function (75, 76) as well as LV deformation and dyssynchrony (77) assessment. Moreover, ^123^I-metaiodobenzilguanidine (^123^I-mIBG) imaging represents the reference for non-invasive evaluation of cardiac adrenergic nervous function and has proven useful in the subclinical cardiac damage assessment as well as in the prognostication of patients with chronic heart failure (78). However, similar to the CT, the main limitation of nuclear imaging is radiation exposure, which ranges between 2 and 8 mSv for SPECT imaging (41) and makes the technique less suitable for serial follow-up of patients receiving cardiotoxic therapies.

**FIGURE 6:**
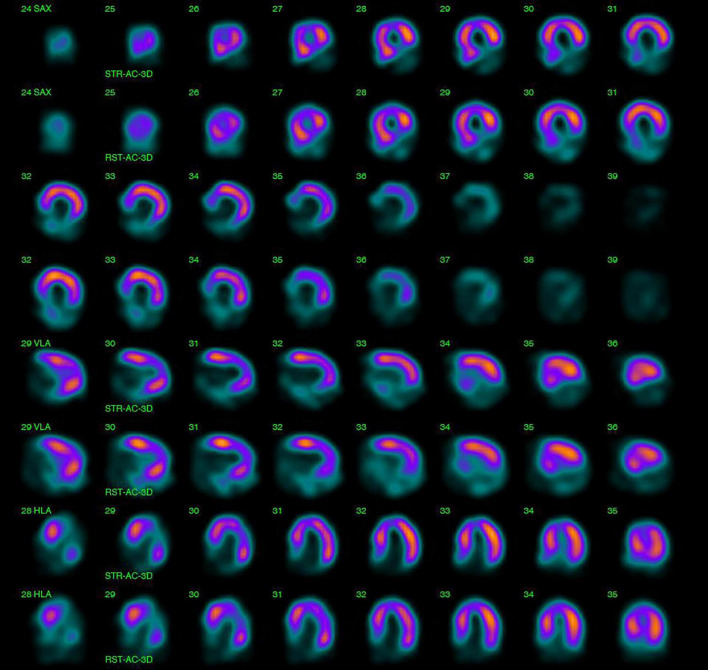
Single-photon emission computed tomography (SPECT) for the assessment of myocardial ischemia and viability. 47-year-old male with a history of Hodgkin lymphoma, treated with radiotherapy and chemotherapy, was diagnosed with regional wall motion abnormalities in the inferior left ventricular wall during regular echocardiographic surveillance and was referred for SPECT. A fixed perfusion defect in the basal and mid inferoseptal, inferior and inferolateral left ventricular segments was found on SPECT with a summed stress and rest score equal to 16 (summed difference score 0). Findings were consisted with a silent myocardial infarction, characterized by non-viable myocardium in the right coronary artery perfusion territory.

Myocardial perfusion imaging with SPECT can provide valuable information on inducible ischemia and myocardial viability (Figure 6). A 14–60% incidence of inducible or new resting perfusion defects with SPECT has been reported in patients with Hodgkin lymphoma, breast and esophageal cancer receiving RT (79–81). Interestingly, a much lower incidence (6–10%) of wall motion abnormities has been described in the same patient cohorts (80, 82). When correlated with coronary angiography findings in Hodgkin lymphoma survivors, perfusion defects with SPECT were associated with a similar sensitivity and much lower specificity to detect 50% coronary artery vessel stenosis (sensitivity 65%, specificity 11%) compared to wall-motion abnormalities with stress echocardiography (sensitivity 59%, specificity 89%) (80). These high false-positive rates of the perfusion defects with SPECT have been attributed to microvascular dysfunction; however, this has not been confirmed with invasive testing.

Myocardial perfusion imaging with PET, most commonly performed with radiotracers ^82^Ru or ^13^NH_4_, offers several advantages over SPECT, including increased spatial and temporal resolution and reliable quantification of absolute myocardial blood flow. PET with ^18^F- fluorodeoxyglucose allows assessment of myocardial inflammation and viability (83). PET with ^18^F-NaF can identify high-risk coronary atherosclerotic plaques by detecting active calcification processes (84). However, the main disadvantage to wider application of PET is its high cost and low availability, as most of the currently available radiotracers require an on-site cyclotron for their production. With development of new and more stable radiotracers that would target multiple pathophysiological processes, PET offers a great potential for early detection of RT-induced cardiotoxicity.

## Gaps in Knowledge and Future Perspectives

Despite the progress in recent years, our knowledge about RT-induced cardiotoxicity is incomplete. The role of multimodality cardiac imaging to address some of the key questions is discussed in the following section.

### Cardiotoxicity With Modern Radiotherapy Techniques, the Existence of Minimal Safe Dose

Large-population studies involving patients with breast cancer and Hodgkin lymphoma have shown a clear dose-effect relationship with adverse cardiac events with no apparent threshold (3, 85, 86). The risk for coronary heart disease increased by 7–16.5% per each Gy increase in the mean heart dose, both with old and modern RT techniques (3, 85, 86). A 10, 30, 40, and 116% percentage increases in the rate of major coronary events were demonstrated in women with breast cancer and mean radiation dose to the heart < 2, 2–4, 5–9, or > 10 Gy, respectively (3). On the other hand, some of the recent studies with modern cardiac imaging techniques have failed to demonstrate significant myocardial injury, questioning the actual cardiotoxicity of modern RT regimens (27, 28, 69, 71). However, there were several important differences between the outcome and the imaging studies. First, the number of patients included in the outcome studies was between 900 and 2,700 compared to < 100 patients in the imaging studies. Furthermore, patients were followed for at least 10 years in the outcome vs. at maximum 2 years in the imaging studies. Therefore, to provide a clear answer about the cardiotoxicity of modern RT regimens and the existence of a potential minimal safe dose, we need additional studies in which large number of patients with different thoracic malignancies will be followed clinically and with modern imaging techniques for longer periods of time.

### Importance of a Specific Cardiac Substructure Radiation

The RT-induced cardiovascular disease also depends on the type and localization of the thoracic tumor and the dose received by a specific cardiac substructure might be more important for the development of cardiac disease than the mean heart dose (6). In a contemporary study of breast cancer patients treated with RT, the volume of the LV receiving 5 Gy was the most important prognostic dose-volume parameter for the occurrence of acute coronary events (85). Mid and distal left anterior descending coronary artery and distal diagonal branch receive the highest radiation doses with 3DCRT of left-sided breast cancer, and a direct link between these areas and the location of coronary stenoses has been demonstrated (87). Furthermore, several imaging studies have shown that regional myocardial injury correlated with the radiation dose distribution (21, 25, 32, 70). Therefore, future RT studies should focus also on the local cardiotoxic effects.

### Best Imaging Parameter to Detect and Monitor Radiotherapy-Induced Cardiotoxicity

Several studies have tried to elucidate the best imaging parameters to predict cardiotoxicity in patients after chest RT. Armstrong et al. (88) have compared 2D and 3D echocardiography with CMR in 114 adult survivors of childhood cancer at a median of 28 years after the exposure to anthracycline chemotherapy and/or chest RT. Mean LVEF with 2D echocardiography was on average 5% higher than with CMR and 3D echocardiography. CMR demonstrated better sensitivity to detect LV systolic dysfunction, i.e., the sensitivity for detection of LVEF < 50% was only 25% with 2D echocardiography and 53% with 3D echocardiography (using CMR as a reference standard). The authors concluded that CMR might be indicated for additional cardiac assessment in high-risk cancer survivors, i.e., patients with LVEF 50–59% on 2D echocardiography. Lambert et al. (19) studied the feasibility of several echocardiographic (2D and 3D LVEF, GLS, global circumferential strain) and CMR (LVEF, GLS, and global circumferential strain with feature-tracking) parameters to detect cardiac dysfunction in breast cancer patients treated with chemotherapy and RT. The 3D LVEF and GLS with echocardiography as well as CMR-derived LVEF showed the largest temporal changes in patients who developed cancer therapeutics-related cardiac dysfunction compared with cancer patients without cardiac dysfunction and healthy volunteers (absolute changes in 2D GLS: 1.9, 0.7, and 0.8%; 3D LVEF: 5.2, 2.3, and 1.8%; and CMR LVEF 6.6, 2.7, and 2.2%, respectively). These 3 parameters also had the lowest interobserver and intraobserver variability, making them most suitable for clinical application. Houbois et al. (89) studied the prognostic value of different echocardiographic and CMR strain parameters. 2D GLS provided the highest discriminatory value over baseline clinical risk factors for subsequent cancer therapeutics-related cardiac dysfunction and was recognized as the optimal prognostic parameter with 2D echocardiography. In addition, GLS with feature-tracking CMR provided an additional prognostic value to CMR-derived LVEF (89). On the other hand, Altaha et al. (90) questioned the usefulness of CMR parametric imaging, since the temporal variability for native T1, T2, and ECV values in cancer patients who developed cancer therapeutics-related cardiac dysfunction was similar to the temporal variability in healthy volunteers.

While measuring LVEF and GLS currently represents a cornerstone in detecting and monitoring RT-induced cardiac toxicity (16, 17), future studies should provide new insights about the most sensitive and reliable imaging parameters. Particularly, the role of CMR parametric imaging should be elucidated as the T1, T2, and ECV allow us to monitor tissue processes like edema, inflammation and fibrosis, which are the fundamental processes in RT-induced cardiac toxicity.

### Combined Value of Imaging, Serum, and Genetic Biomarkers

In addition to imaging biomarkers, blood-based and genetic biomarkers could help identify patients at higher risk for cardiovascular complications. Classic cardiac biomarkers such as N-terminal pro b-type natriuretic peptide, troponin I and T have been linked to RT-induced cardiotoxicity (21, 91–93). Several other serum biomarkers, involved in oxidative stress, inflammation, and vascular remodeling, have also been studied in patients receiving cancer treatments (92–94) and circulating microRNAs and extracellular vesicles are tested in ongoing clinical trials (95). Furthermore, the association between genetic polymorphisms and the risk of developing cardiovascular disease after chest RT has been demonstrated (96, 97). However, the knowledge about the role of genetic variability for the occurrence of RT-induced cardiotoxicity is limited and further studies are needed in this field (98). Different biomarkers may reflect different cardiac complications of RT and the integration of serum/genetic and imaging biomarkers might serve as a better predictor of RT-induced cardiotoxicity.

### Ongoing Clinical Trials

Several ongoing clinical trials aim to close the gaps in our current knowledge about RT-induced cardiotoxicity. The CareBest is a single-center prospective clinical trial, which will enroll over 2,000 breast cancer patients undergoing chemotherapy and RT (99). The study protocol includes CMR scanning at 3, 6, and 24 months after treatment and the patients will be followed for 4 years. The primary objective is to evaluate the prognostic value of different CMR parameters (including T1 mapping and strain) to predict clinical outcome (major adverse cardiac events). In addition, temporal relationships between myocardial injury and functional consequences will be studied according to different treatment regimens (e.g., the choice of chemotherapeutic agents, targeted therapy agents, and radiation dose). Another single-center prospective clinical trial will enroll 60 patients with thoracic cancer (breast, esophagus, or lung) undergoing curative chest RT with or without chemotherapy (100). Early cardiotoxicity (1 and 12 weeks after RT) will be studied with speckle-tracking echocardiography, CMR and serum biomarkers. The MEDIRAD EARLY HEART is a multi-center, prospective cohort study including 250 breast cancer patients treated with primary breast-conserving surgery and RT without neoadjuvant or adjuvant therapy (95). The RT-induced cardiotoxicity will be studied with speckle-tracking echocardiography, CMR, coronary CTA, and circulating biomarkers (including novel markers like extracellular vesicles and microRNA) at 6 months and 2 years after RT. We may reasonably assume that these studies will answer some of the questions raised in this section.

## Conclusion

Cardiovascular imaging has shown remarkable progress in the developing field of cardio-oncology, providing highly sensitive methods for diagnosing cardiotoxicity. LVEF, preferably with 3D echocardiography or CMR, and GLS with speckle-tracking echocardiography are currently the recommended parameters to detect RT-induced cardiotoxicity (16, 17). However, several novel imaging parameters, as well as serum and genetic biomarkers are tested in the ongoing clinical trials. One of the key questions that remains to be elucidated is whether meticulous monitoring with cardiac imaging improves the outcome of cancer patients and prevents late complications. Data from the chemotherapy trials do not support routine administration of angiotensin receptor blockers or beta blockers to prevent heart failure (101). Therefore, future studies should try to identify the subgroups of patients at high risk for cardiotoxicity who may benefit from cardioprotective therapies. Integration of clinical, dosimetric, molecular factors and multimodality cardiac imaging in multifactorial models may provide a platform for individualized precision-based medical care.

## Author Contributions

TP, BB, JD, KG, and TM involved in drafting and writing the manuscript. All coauthors provided final approval of the submitted manuscript.

## Conflict of Interest

The authors declare that the research was conducted in the absence of any commercial or financial relationships that could be construed as a potential conflict of interest.

## Publisher’s Note

All claims expressed in this article are solely those of the authors and do not necessarily represent those of their affiliated organizations, or those of the publisher, the editors and the reviewers. Any product that may be evaluated in this article, or claim that may be made by its manufacturer, is not guaranteed or endorsed by the publisher.

## Abbreviations

2D: two-dimensional
3D: three-dimensional
3DCRT: three-dimensional conformal radiotherapy
CAC: coronary artery calcium
CMR: cardiovascular magnetic resonance
CT: computed tomography
CTA: computed tomography angiography
ECV: extracellular volume
GLS: global longitudinal strain
HU: Hounsfield units
IMRT: intensity-modulated radiotherapy
LV: left ventricle/left ventricular
LVEF: left ventricular ejection fraction
PET: positron emission tomography
RT: radiotherapy
RV: right ventricle/right ventricular
SPECT: single-photon emission computed tomography.
